# Injury risk in British Columbia, Canada, 1986 to 2009: are Aboriginal children and youth over-represented?

**DOI:** 10.1186/s40621-015-0039-2

**Published:** 2015-05-21

**Authors:** M Anne George, Andrew Jin, Mariana Brussoni, Christopher E Lalonde, Rod McCormick

**Affiliations:** 1 Department of Pediatrics, University of British Columbia, Vancouver, British Columbia Canada; 2 Child & Family Research Institute, Vancouver, British Columbia Canada; 3 School of Population & Public Health, University of British Columbia, Vancouver, British Columbia Canada; 4 Epidemiology Consultant, Surrey, British Columbia Canada; 5 Department of Psychology, University of Victoria, Victoria, British Columbia Canada; 6 Faculty of Human, Social, and Educational Development, Thompson Rivers University, Kamloops, British Columbia Canada; 7 Faculty of Medicine, University of British Columbia, Room 9-387, 3333 University Way, Prince George, BC V2N-4Z9 Canada

**Keywords:** Wounds and injuries (MeSH), Indian, North American (MeSH), Indigenous population (MeSH), Aboriginal, First Nations, British Columbia (MeSH), Canada (MeSH), Epidemiology (MeSH), Children (MeSH), Adolescent (MeSH)

## Abstract

**Background:**

Children and youth worldwide are at high risk of injury resulting in morbidity, disability or mortality. Disparities in risk exist between and within countries, and by sex and ethnicity. Our aim is to contribute data on disparities of injury rates for Aboriginal children and youth compared with those of the general population in British Columbia (BC), Canada, by examining risks for the two populations, utilizing provincial administrative data over a 24-year period.

**Methods:**

Hospital discharge records from the provincial health care database for children and youth were used to identify injury for the years 1986 to 2009. Within the total BC population, the Aboriginal population was identified. Crude rates and standardized relative risks (SRR) of hospitalization were calculated, by year and category of injury type and external cause, and compared to the total BC population for males and females under age 25 years.

**Results:**

Over the 24-year period, substantive decreases were found in hospitalization injury risks for children and youth in both Aboriginal and total populations, for both sexes, and for most categories and types of injuries. Risk in overall injury dropped by 69% for the Aboriginal population and by 66% for the total BC population, yet in every year, the Aboriginal population had a higher risk than the total BC population. There were over 70% declines in risks among females of intentionally inflicted injury by another, among both the Aboriginal and total BC populations. Risk of injury caused by transport vehicles has decreased by an overwhelming 83% and 72% for the Aboriginal male population and for the total BC male population, respectively.

**Conclusions:**

The over 70% declines in risks for females of intentionally inflicted injury by another, among both the Aboriginal and total BC populations is excellent news. Risk of injury caused by transport vehicles for males decreased overwhelmingly for both populations. Disparities in rates between the Aboriginal population and total BC population remain because of similarity in the proportional reductions among the two populations. Since the Aboriginal population started at a much higher risk, in absolute terms, the gap between the two populations is shrinking.

## Background

Children and youth worldwide are at high risk of injury that results in morbidity, disability or mortality (Gore et al. 2011; Laflamme et al. 2010, WHO World Health Organization 2008; WHO 2002). Over 40% of the world’s injury related mortality occurs in people under age 30 years (LaFlamme et al. 2010). Promisingly, child and youth injury rates have been declining in some higher income countries, such as the United States (USA) (US Department of Health and Human Services, 2014); Sweden (WHO 2008) and Canada (Pan et al. 2007; Harrop et al. 2007; Pan et al. 2006; Birken et al. 2006; Harrop et al. 2007). This is not the case in low and middle income countries, which has the largest burden, partially due to war, and where the burden is expected to rise dramatically over the next few years as rates of motorization increase (WHO 2008; WHO 2002).

Downward trends in child and youth injury rates may be slowing, at least in the USA (US Department of Health and Human Services, 2014). In the USA, injury rates for emergency room visits declined overall from 1980 to 2010 for children under age 18 years; however, rates have leveled off or even increased slightly since 2005 (US Department of Health and Human Services, 2014).

The overall impressive decreases in injury rates mask disparities by gender, socioeconomic background and ethnicity (Mytton et al. 2012). Boys are consistently shown to be at higher risk than girls (Gore et al. 2011; Oliver and Kohen 2010; Moorin and Hendrie 2008; Ekman et al. 2005; Spady et al. 2004). The decline in overall population injury fatalities is unevenly distributed across genders and municipalities in Sweden (Ekman et al. 2005), and injury rate disparities according to socioeconomic background have also been reported for children in Canada (Oliver and Kohen 2010; Pan et al. 2006; Spady et al. 2004), in the United Kingdom (Brownell et al. 2010), and in countries worldwide (WHO 2002). Similarly in Australia, the decreasing trends in rates of injury related morbidity and mortality are not evenly distributed across the population (Moorin and Hendrie 2008). For the youngest Australians, children aged 0–4, injury hospitalization rates decreased during the period 1999 to 2000 to 2010 to 2011; but these data reflect an increase in rates for males and a decrease in rates for females (Australian Institute of Health and Welfare 2013).

Ethnicity is a predictor of child and youth injury rates worldwide, with indigenous children being at significantly higher risk for morbidity and mortality from unintentional injuries Möller et al. 2015). Möller et al. (2015) found consistently higher rates for indigenous children in nearly all 39 studies included in their systematic review, across countries, age groups and types and causes of injury, noting that four Canadian studies reported the greatest differences in between populations of indigenous and non-indigenous children.

The four Canadian studies (Peters et al. 2013; Oliver et al. 2012; Oliver and Kohen 2012; Alaghehbandan et al. 2010) described by Möller et al. (2015) used population data from the provincial universal health care insurance programs, but faced challenges in accurately identifying Aboriginal populations. Oliver and Kohen (2012) who identified indigenous populations according to proportion of Aboriginal populations in the residential area, found unintentional injury hospitalization rates for children and youth to be at least twice as high in areas with a high percentage of people identifying as Aboriginal compared to areas with low percentage of identifying Aboriginal people. For some injury types; for example, drowning/suffocation, rates were three times higher in the high Aboriginal identity areas. Studies of populations of Indian or First Nations reserves or other geographic areas with a high proportion of indigenous residents, and that make age and gender-standardized comparisons with the general population, are biased, because (1) they exclude indigenous people who live off-reserve or in urban areas, where injury rates are lower, and (2) they do not take into account the effects of northern or rural environments on injury rates.

We sought to improve estimates of the disparities in unintentional injury rates for Aboriginal children and youth compared with those of the general population in the Canadian province of British Columbia (BC). The current study uses population-based administrative data of injuries over a 24-year period of 1986 to 2009. We employed a method adapted from BC Vital Statistics (British Columbia Vital Statistics Agency 2004) using the province’s universal health care insurance program as a population registry, and identified Aboriginal people within the population by record linkage, using a combination of insurance premium group, Indian status, and birth and death record notations. In adapting this method, two improvements were made; firstly, a broader range of injury events was studied by using hospitalization data instead of mortality data, providing opportunities to examine variability in rates by smaller categories of injury type and cause, demographic groups, and time periods; and secondly, compensation was made for the effects of northern and rural locations by standardized comparisons of injury risks between the Aboriginal and general populations, by age, gender and also region of the province. Other than our own studies (George et al. 2015; Brussoni et al. 2014, Jin et al. 2014, George et al. 2013) and the study from which our method was adapted (British Columbia Vital Statistics Agency 2004), no other Canadian studies of injury among children and youth using this method were found.

## Methods

The University of British Columbia Behavioural Research Ethics Board reviewed and approved the methods for this study. The Data Stewards representing the BC Ministry of Health Services and the Vital Statistics Agency of BC approved the data access request. We used existing databases, permanently linked by British Columbia Personal Health Number, maintained by Population Data BC (BC Ministry of Health 2011, 2012; BC Vital Statistics Agency 2011a, 2011b). Population Data BC rendered the client records anonymous before our analysis.

As described elsewhere (George et al. 2015; Brussoni et al. 2014; Jin et al. 2014) we used the BC Medical Services Plan (MSP), the province’s universal health care insurance program, as a registry of the total resident population of BC. One-day extracts of the consolidated registration and premium billing files of the MSP at the mid-points of the fiscal years 1985 to 86 through 2008 to 2009 (British Columbia Ministry of Health 2012) were obtained. Within that population, we identified as Aboriginal any person with:

- Membership in MSP Premium Group 21, which identifies that the insurance premiums were paid by First Nations and Inuit Health Program, Health Canada, for reason of Aboriginal status, OR
- One or both parents with Aboriginal status or resident on an Indian Reserve, as indicated on the linked Vital Statistics birth record (British Columbia Vital Statistics Agency 2011a), OR
- Aboriginal status or resident of a First Nation reserve, as indicated on the linked Vital Statistics death record (British Columbia Vital Statistics Agency 2011b).

We previously described this method, and discussed the quality of the population registry, and validity and limitations of the Aboriginal identification (George et al. 2015, Jin et al. 2015). The basis of our definition of “Aboriginal” is the legal recognition of Indian status, though indirectly, because we could not obtain direct access to the Indian Status Registry for privacy reasons. This indirect method has the advantage of including children who are eligible for Indian status because of their parents’ Indian status, but who themselves have not yet applied for Indian Status. Family accounts are included in MSP Premium Group 21 if the account’s primary registrant declares Indian status. Some people with Indian status who are eligible to join MSP Premium Group 21 might not do so because another party (e.g., an employer) pays their health insurance premiums. Out of an interest in avoiding payment for these premiums, other parties try to identify people with Indian status. When the Vital Statistics Agency of BC used the same method as ours but included additional people found only in the Indian Status Registry, their count was 151,783 Aboriginal persons in BC in 2002 (BC Vital Statistics Agency 2004), compared to our count of 135,076 that year. In the 2006 Census, 196,070 residents of BC identified themselves as “an Aboriginal person, that is, North American Indian, Métis or Inuit (Eskimo),” compared to our count of 148,458 persons who met our definition of Aboriginal in 2006. The restrictiveness of our definition protects the internal validity of our analysis since any undercounting applies to both the numerator (hospitalization counts) and the denominator (population counts). Because we identified hospitalizations as Aboriginal or not by linking to our population registry, there should be no bias in our calculated rates of hospitalization among Aboriginal people.

Population counts were tabulated according to year, gender, five-year age group, Aboriginal status, and Health Service Delivery Area (HSDA), of which there are 16 in BC.

Hospitalization counts were calculated from discharge summary records representing hospital separations occurring in BC from January 1, 1986 through December 31, 2009 (British Columbia Ministry of Health 2012). To be counted, hospitalizations must have been of at least one day duration. Day surgery hospitalizations of less than one day, and emergency room visits, regardless of duration, were not counted. Hospitalizations were considered to be “due to injury” if the level of care was “acute” or “rehabilitation”, and the Most Responsible Diagnosis on the discharge record was an International Classification of Diseases Revision 9 (ICD 9) code in the range 800 through 999, or an International Classification of Diseases Revision 10 (ICD 10) code in the range S00 through T98. Injury hospitalization *type* (trauma, poisoning, burn or other) was classified using Most Responsible Diagnosis codes. Starting April 1, 1991, discharge records included supplemental diagnosis codes (ICD 9 codes E800 through E999, or ICD 10 codes V01 through Y98), describing injuries by intention (unintentional, intentionally inflicted by self, or intentionally inflicted by another) and by external cause (transportation, medical/surgical mishap, falls, poison, fire, etc.). Hospitalizations were classified by intention and external *cause* using the first occurrence of a supplemental injury diagnosis code, since some injury hospitalizations had more than one supplemental code. Injury classification categories and the associated diagnostic codes are shown in Table 1.

**Table 1:** **Injury categories derived from International Classification of Diseases codes**

**Injury category**	**ICD 9 codes**	**ICD10 codes**
**Injury types**		
All injury types	800 to 999	S00 toS99, T00 to T98
Trauma	800 to 908, 910 to 939, 950 to 959	S00 to S99, T00 to T19, T79, T90 to T94
Poisoning	909.0, 909.1, 960 to 989	T36 to T65, T96 to T97
Burn	940 to 949	T20 to T32, T95
Other injury types	909.2 to 909.9, 990 to 999	T33 to T35, T66 to T78, T80 to T88, T98
Injury causes		
Unintentional injury	E800 to E928, E930 to E949	V01 to X59, Y40 to Y84
Transport vehicle (unintentional)	E800 to E807, E810 to E829, E831, E833 to E838, E840 to E848	V01 to V89, V91, V93 to V99
Medical/surgical mishaps	E870 to E876, E878 to E879, E930 to E949	Y40 to Y84
Falls (unintentional)	E880 to E888	W00 to W19
Self-inflicted injury	E950 to 958	X60 to X84
Poisoning (self-inflicted)	E950 to E952	X60 to X69
Intentionally inflicted by another	E960 to E968	X85 to Y09

Numbers of injury hospitalizations were tabulated by injury type, injury intention and external cause category, calendar year of discharge, gender, 5-year age group, Aboriginal status, and HSDA of residence.

Among persons aged 25 years and under, we calculated the crude rate of hospitalization as the number of hospital separations divided by the person-years of observation (the sum of the annual population counts) during the same time period. We considered the crude rate to be a binomial proportion, and estimated standard errors of the proportion, and 95% confidence intervals of the proportion accordingly. We calculated standardized relative risk (SRR) of hospitalization relative to the risk of hospitalization in the reference population (the combined under 25 years of age population of BC during the specified observation period: 30,370,743 person-years from 1986 to 2009, or 24,690,923 person-years from 1991 to 2009) using the method of indirect standardization (Kahn and Sempos 1989), standardizing by gender, 5-year age group, and HSDA. We took the gender, age and HSDA specific rates of hospitalization in the reference population, and multiplied them by the person-year counts within the corresponding cells of the target population, summing to the indirectly standardized expected number of hospitalizations in the target population. We considered the expected rate (the expected number divided by the number of person-years) to be a binomial proportion, and we estimated standard errors and 95% confidence intervals accordingly. The standardized relative risk (“SRR”) (relative to the total population of BC) is the crude rate of hospitalizations divided by the expected rate of hospitalizations. Since these rates have the same denominator (person-years) the SRR simplifies to the observed number of hospitalizations divided by the expected number of hospitalizations. This is analogous to the Standardized Mortality Ratio (if death is the event counted), and could also be called the Standardized Incidence Ratio.

Note that the reference population is the combined total population under age 25 years in BC during the entire observation period; therefore, for the total population of BC, the SRR in a particular year can be higher or lower than one, but the average of the SRRs over all the years will be one.

Some might argue that the reference population should have been the *non-Aboriginal* population of BC, but with the indirect standardization method, the comparison is between the observed number or rate in the population of interest (i.e., Aboriginal, a small population with unstable rates) and the expected number or rate in the reference population (a large population with stable rates). It would be customary, and preferable to use the total population (rather than the non-Aboriginal population) as the reference population, because the total population is larger and has more stable rates. In this case the difference between the non-Aboriginal and total populations would not be material, because non-Aboriginal people are more than 95% of the total population under 25 years of age.

Cumulative change in SRR was assessed over time as the relative change between the first and last years of the observation period, i.e., (SRR_2_/SRR_1_) - 1. To facilitate comparisons, we converted relative change over a period of multiple years to an annualized change, using the following formula.

$$ {\left(\frac{SR{R}_2}{SRR{}_1}\right)}^{1/\left({t}_2-{t}_1\right)}-1 $$

**Figure 1:**
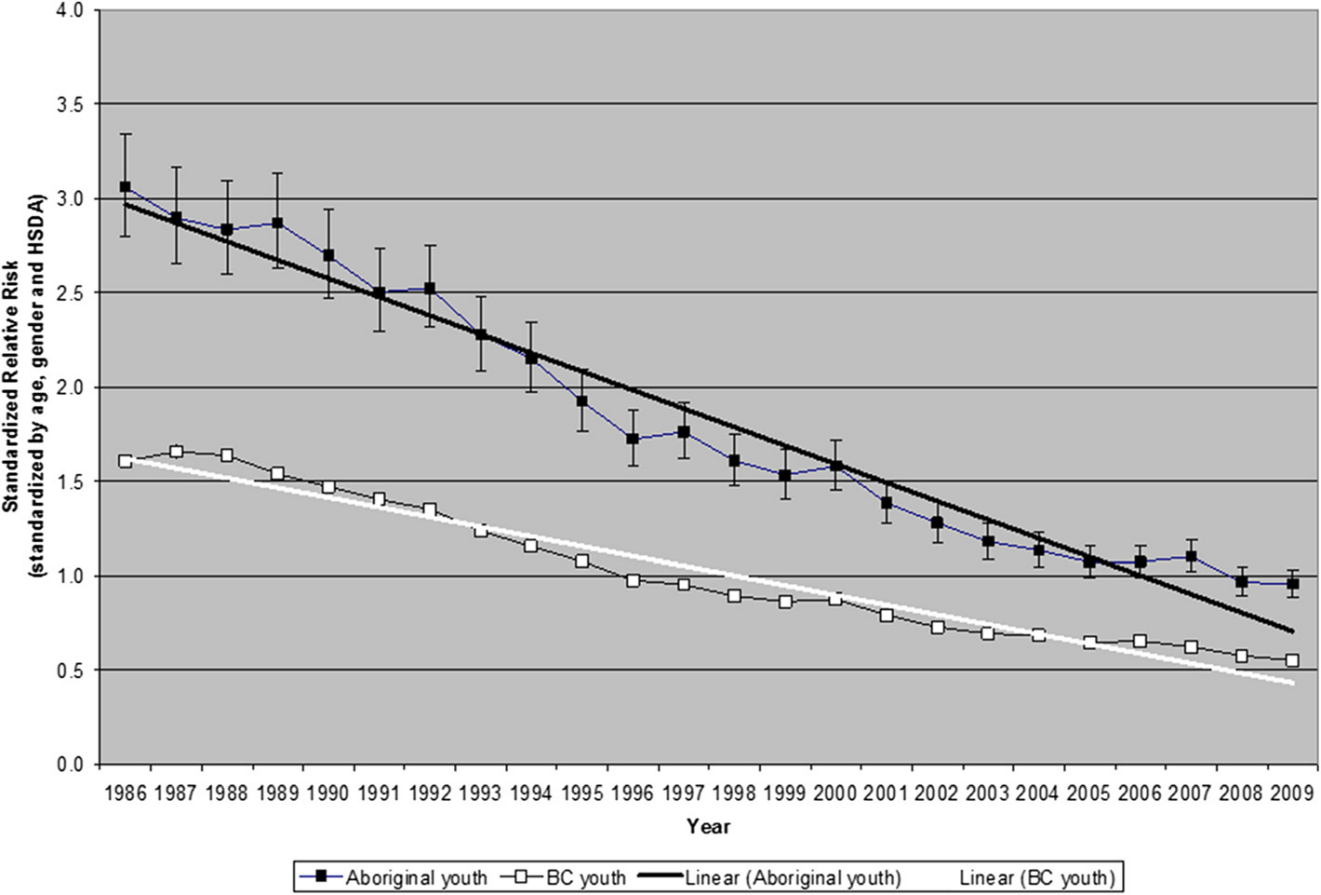
Standardized relative risk of Injury, British Columbia, 1986 to 2009, under 25 years of age.

We compared the cumulative change (from the first to the last years) among Aboriginal people under 25 years of age to the cumulative change over the same period among the total population of BC under 25 years of age. We tested the statistical significance of the difference by calculating the probability (2-sided, z-test) that Ln((SRR_2_)/(SRR_1_)) Aboriginal = Ln((SRR_2_)/(SRR_1_)) BC. For illustrative purposes, we plotted regression lines of SRR as a function of year (see Figure 1), but we did not attempt to test the statistical significance of the difference between the slope of the Aboriginal line and the slope of the total population of BC line, because in a time series the values of the dependent variable (SRR) are not independent of each other (they are autocorrelated, thus violating one of the fundamental assumptions of linear regression) and therefore linear regression does not provide valid estimates of the standard errors of the slopes.

## Results and discussion

As we reported previously (Jin et al. 2015) injury crude rates and SRRs were highly variable among HSDAs, and higher among northern and non-metropolitan HSDAs. Standardizing by HSDA reduced the disparity between the Aboriginal and total populations, because Aboriginal people are more likely to reside in northern or non-metropolitan regions where injury rates are higher. Having standardized by HSDA (in addition to age and gender), we know the disparities, and the changes over time, are real and not due to geographic or demographic redistributions of the populations over time.

Crude rates and SRRs of hospitalizations due to all injuries under age 25 years, from 1986 to 2009, are shown in Table 2 and Figure 1, for the Aboriginal population and for the total population of BC, by year. In every year, the Aboriginal population had a higher crude rate and higher SRR of injury hospitalization than did the total BC population (the 95% confidence intervals do not overlap) and for most years a reduction in rate was observed for each population.

**Table 2:** **Hospital separations due to injury [1], British Columbia, age under 25 years, 1986 to 2009 [2], by calendar year**

	**Aboriginal population**					**Total population of BC**				
**Year**	**Obs [3]**	**Rate [4]**	**95% CI for Rate**	**SRR [5]**	**95% CI for SRR**	**Obs [3]**	**Rate [4]**	**95% CI for Rate**	**SRR [5]**	**95% CI for SRR**
1986	1,501	282	268 to 296	3.06	2.80 to 3.34	14,106	124	122 to 126	1.61	1.57 to 1.64
1987	1,433	267	253 to 280	2.90	2.65 to 3.16	14,241	126	124 to 128	1.66	1.62 to 1.69
1988	1,411	260	247 to 273	2.83	2.60 to 3.09	13,865	124	122 to 126	1.64	1.60 to 1.67
1989	1,439	262	249 to 276	2.87	2.63 to 3.13	13,075	115	113 to 117	1.54	1.51 to 1.57
1990	1,347	245	233 to 259	2.70	2.47 to 2.94	12,648	109	108 to 111	1.47	1.44 to 1.50
1991	1,266	226	214 to 239	2.50	2.30 to 2.73	12,212	104	102 to 106	1.40	1.37 to 1.43
1992	1,286	227	215 to 239	2.52	2.31 to 2.75	11,983	100	98 to 102	1.35	1.32 to 1.38
1993	1,185	204	192 to 215	2.28	2.09 to 2.48	11,330	92	90 to 93	1.24	1.21 to 1.26
1994	1,129	190	180 to 202	2.15	1.98 to 2.34	10,868	86	84 to 87	1.15	1.13 to 1.18
1995	1,017	169	159 to 180	1.92	1.77 to 2.09	10,279	80	78 to 81	1.08	1.05 to 1.10
1996	925	152	142 to 162	1.72	1.59 to 1.88	9,495	72	71 to 74	0.98	0.96 to 1.00
1997	947	155	145 to 165	1.76	1.62 to 1.92	9,368	71	69 to 72	0.95	0.93 to 0.97
1998	866	141	132 to 151	1.61	1.48 to 1.75	8,797	66	65 to 68	0.89	0.88 to 0.91
1999	826	134	126 to 144	1.53	1.41 to 1.67	8,478	64	63 to 66	0.86	0.85 to 0.88
2000	857	138	129 to 147	1.58	1.45 to 1.72	8,533	65	64 to 67	0.88	0.86 to 0.89
2001	762	121	112 to 130	1.38	1.27 to 1.50	7,732	59	58 to 60	0.79	0.78 to 0.81
2002	715	111	103 to 120	1.28	1.18 to 1.39	7,133	54	53 to 56	0.73	0.71 to 0.74
2003	693	103	96 to 111	1.18	1.09 to 1.28	6,907	52	51 to 53	0.69	0.68 to 0.71
2004	679	99	92 to 107	1.13	1.05 to 1.23	6,835	51	50 to 53	0.68	0.67 to 0.70
2005	655	94	87 to 101	1.07	0.99 to 1.16	6,457	49	47 to 50	0.65	0.63 to 0.66
2006	667	94	87 to 101	1.07	0.99 to 1.16	6,497	49	48 to 50	0.65	0.64 to 0.67
2007	698	96	89 to 103	1.10	1.02 to 1.19	6,191	47	46 to 48	0.62	0.61 to 0.63
2008	623	84	77 to 91	0.97	0.89 to 1.04	5,740	43	42 to 44	0.58	0.56 to 0.59
2009	629	83	77 to 90	0.96	0.89 to 1.03	5,503	41	40 to 42	0.55	0.54 to 0.56
1986 to 2009	23,556	158	156 to 160	1.78	1.75 to 1.81	228,273	75	75 to 75	1	[reference]

Notes:

1. "Injury" defined as Most Responsible Diagnosis in the range ICD9:800 to 999, or ICD10:S00 to T98.

2. Separations occurring during the observation period 1 January 1986 to 31 December 2009.

3. Observed number of hospital separations (acute or rehabilitation care).

4. Crude Rate per 10,000 person-years.

5. Standardized relative risk compared to total population of BC, standardized by age, gender and HSDA.

Table 3 shows crude rates and SRRs of hospitalizations due to injuries during the first year (1986 or 1991) and last year (2009) of the observation period, among the Aboriginal population and the total BC population, by gender and major categories of injury. Males had higher crude rates of injury hospitalization than did females in both 1986 and 2009. For both genders, a substantial reduction in crude rates was shown between the two time periods among both the Aboriginal population and the total populations of BC.

**Table 3:** **Hospital separations due to injury [1], British Columbia, age under 25 years, 1986 compared to 2009, and 1991 compared to 2009, by injury category**

		**Aboriginal population**					**Total population of BC**				
**Injury category**	**Year**	**Obs [2]**	**Rate [3]**	**95% CI for Rate**	**SRR [4]**	**95% CI for SRR**	**Obs [2]**	**Rate [3]**	**95% CI for Rate**	**SRR [4]**	**95% CI for SRR**
Total, All injuries	1986	1,501	282	268 to 296	3.06	2.80 to 3.34	14,106	124	122 to 126	1.61	1.57 to 1.64
Total, All injuries	2009	629	83	77 to 90	0.96	0.89 to 1.03	5,503	41	40 to 42	0.55	0.54 to 0.56
Male; Total, All injuries	1986	842	322	302 to 345	2.81	2.51 to 3.14	9,150	160	157 to 163	1.66	1.61 to 1.70
Male; Total, All injuries	2009	370	95	86 to 105	0.88	0.80 to 0.97	3,591	53	51 to 54	0.55	0.54 to 0.57
Female; Total, All injuries	1986	655	243	225 to 262	3.48	3.02 to 4.01	4,895	87	85 to 90	1.54	1.48 to 1.59
Female; Total, All injuries	2009	259	70	62 to 79	1.08	0.95 to 1.23	1,912	29	28 to 31	0.55	0.53 to 0.57
Trauma	1986	1,109	208	196 to 221	2.99	2.70 to 3.31	11,271	99	97 to 101	1.68	1.64 to 1.72
Trauma	2009	418	55	50 to 61	0.84	0.77 to 0.92	4,030	30	29 to 31	0.53	0.52 to 0.54
Poisoning	1986	238	45	39 to 51	3.87	3.01 to 4.97	1,380	12	11 to 13	1.39	1.30 to 1.47
Poisoning	2009	108	14	12 to 17	1.38	1.11 to 1.73	659	5	5 to 5	0.58	0.55 to 0.62
Burn	1986	63	12	9 to 15	4.89	2.82 to 8.67	422	4	3 to 4	1.93	1.69 to 2.21
Burn	2009	17	2	1 to 4	0.91	0.58 to 1.45	114	1	1 to 1	0.50	0.44 to 0.57
Other injury type	1986	91	17	14 to 21	2.00	1.49 to 2.68	1,033	9	9 to 10	1.19	1.11 to 1.27
Other injury type	2009	86	11	9 to 14	1.36	1.06 to 1.74	700	5	5 to 6	0.69	0.65 to 0.74
Unintentional injury	1991	767	183	170 to 196	2.82	2.51 to 3.18	8,366	95	93 to 97	1.75	1.70 to 1.80
Unintentional injury	2009	470	62	57 to 68	0.98	0.90 to 1.07	4,641	35	34 to 36	0.64	0.63 to 0.66
Transport vehicle (unintentional)	1991	262	62	55 to 70	3.29	2.64 to 4.10	2,743	31	30 to 32	2.06	1.95 to 2.17
Transport vehicle (unintentional)	2009	89	12	10 to 14	0.65	0.55 to 0.77	1,146	9	8 to 9	0.54	0.52 to 0.56
Medical/surgical mishaps	1991	58	14	11 to 18	1.92	1.34 to 2.76	730	8	8 to 9	1.27	1.17 to 1.37
Medical/surgical mishaps	2009	71	9	7 to 12	1.32	1.01 to 1.73	627	5	4 to 5	0.71	0.67 to 0.76
Falls (unintentional)	1991	231	55	48 to 63	2.71	2.19 to 3.35	2,501	28	27 to 30	1.60	1.52 to 1.68
Falls (unintentional)	2009	158	21	18 to 24	1.03	0.88 to 1.21	1,504	11	11 to 12	0.67	0.64 to 0.69
Self-inflicted injury	1991	122	29	24 to 35	4.53	3.10 to 6.66	663	8	7 to 8	1.59	1.45 to 1.75
Self-inflicted injury	2009	62	8	6 to 10	1.45	1.08 to 1.97	401	3	3 to 3	0.58	0.54 to 0.62
Poisoning (self-inflicted)	1991	97	23	19 to 28	4.12	2.75 to 6.23	574	7	6 to 7	1.54	1.39 to 1.70
Poisoning (self-inflicted)	2009	56	7	6 to 10	1.50	1.09 to 2.08	370	3	3 to 3	0.59	0.55 to 0.64
Intentionally inflicted by another	1991	94	22	18 to 27	5.10	3.22 to 8.16	447	5	5 to 6	1.47	1.31 to 1.64
Intentionally inflicted by another	2009	74	10	8 to 12	2.39	1.68 to 3.42	376	3	3 to 3	0.71	0.65 to 0.77

Notes:

1. "Injury" defined as Most Responsible Diagnosis in the range ICD9:800 to 999, or ICD10:S00 to T98.

2. Observed number of hospital separations (acute or rehabilitation care).

3. Crude Rate per 10,000 person-years.

4. Standardized relative risk, compared to the total population of BC, standardized by age, gender and HSDA.

In every category of injury, the Aboriginal population had a higher crude rate and higher SRR of injury hospitalization than did the total BC population, at both the start and the end of the observation period. These tables also show the substantial reductions for both populations in every category of injury in crude rates and standardized risks, between 1986 and 2009, or between 1991 and 2009 for injuries categorized by intent and external cause.

Table 4 shows relative changes in SRR between 1986 and 2009 among the Aboriginal and total BC populations, by gender and major categories of injury type. Table 5 shows relative changes in SRR between 1991 and 2009, by gender and major categories of injury. Over these observation periods, there were substantial decreases for Aboriginal and total BC populations, for both genders, and for all major categories of injury. The relative decreases were not statistically significantly different comparing the Aboriginal population to the total population of BC; however, making gender-specific comparisons between the two populations, two statistically significant differences emerged.

**Table 4:** **Standardized relative risks of hospitalization due to injury (categorized by type), British Columbia, age under 25 years, 1986 to 2009**

**Population; Injury type**	**SRR 1986**	**SRR 2009**	**1986 to 2009 % change**	p*	**Annual % change**	**L95CL**	**U95CL**
Aboriginal							
Total, All injuries	3.06	0.96	−68.8%	0.119	−4.7%	−5.2%	−4.3%
Trauma	2.99	0.84	−72.0%	0.100	−5.2%	−5.7%	−4.6%
Poisoning	3.87	1.38	−64.2%	0.365	−4.2%	−5.5%	−2.9%
Burn	4.89	0.91	−81.4%	0.367	−6.8%	−9.5%	−4.0%
Other injury type	2.00	1.36	−32.2%	0.457	−1.6%	−3.2%	0.0%
Aboriginal, Male							
Total, All injuries	2.81	0.88	−68.5%	0.438	−4.7%	−5.3%	−4.1%
Trauma	2.71	0.80	−70.3%	0.439	−4.9%	−5.6%	−4.3%
Poisoning	4.32	1.09	−74.8%	0.238	−5.6%	-7.8%	−3.3%
Burn	4.50	1.13	−74.9%	0.970	−5.6%	−8.9%	−2.2%
Other injury type	1.91	1.40	−26.4%	0.444	−1.3%	−3.4%	0.9%
Aboriginal, Female							
Total, All injuries	3.48	1.08	−69.0%	0.157	−4.8%	−5.5%	−4.0%
Trauma	3.58	0.92	−74.5%	0.153	−5.5%	−6.5%	−4.6%
Poisoning	3.68	1.56	−57.5%	0.724	−3.5%	−5.2%	−1.8%
Burn	6.01	0.37	−93.8%	0.038	−11.0%	−15.8%	−5.9%
Other injury type	2.11	1.30	−38.4%	0.792	−2.0%	−4.2%	0.3%
BC							
Total, All injuries	1.61	0.55	−65.7%		−4.4%	−4.5%	−4.2%
Trauma	1.68	0.53	−68.5%		−4.7%	−4.8%	−4.6%
Poisoning	1.39	0.58	−58.1%		−3.6%	−3.9%	−3.2%
Burn	1.93	0.50	−73.9%		−5.4%	−6.2%	−4.7%
Other injury type	1.19	0.69	−41.5%		−2.2%	−2.6%	−1.8%
BC, Male							
Total, All injuries	1.66	0.55	−66.6%		−4.5%	−4.6%	−4.3%
Trauma	1.70	0.54	−68.3%		−4.7%	−4.8%	−4.5%
Poisoning	1.58	0.57	−64.3%		−4.2%	−4.8%	−3.6%
Burn	2.03	0.52	−74.5%		−5.5%	−6.4%	−4.7%
Other injury type	1.17	0.70	−40.3%		−2.1%	−2.6%	−1.6%
BC, Female							
Total, All injuries	1.54	0.55	−64.3%		−4.2%	−4.4%	−4.0%
Trauma	1.67	0.51	−69.5%		−4.8%	−5.1%	−4.6%
Poisoning	1.28	0.59	−54.1%		−3.2%	−3.6%	−2.8%
Burn	1.78	0.47	−73.7%		−5.4%	−6.8%	−4.0%
Other injury type	1.21	0.69	−43.0%		−2.3%	−2.9%	−1.8%

Notes:

* probability (2-sided, z-test) that Ln((SRR 2009)/(SRR 1986)) Aboriginal = Ln((SRR 2009)/(SRR 1986)) BC.

**Table 5:** **Standardized relative risks of hospitalization due to injury (categorized by cause), British Columbia, age under 25 years, 1991 to 2009**

**Population; Injury cause**	**SRR 1991**	**SRR 2009**	**1991 to 2009 % change**	p*	**Annual % change**	**L95CL**	**U95CL**
Aboriginal							
Unintentional injury	2.82	0.98	−65.3%	0.488	−5.4%	−6.2%	−4.7%
Transport vehicle (unintentional)	3.29	0.65	−80.1%	0.059	−8.2%	−9.5%	−6.8%
Medical/surgical mishaps	1.92	1.32	−31.2%	0.391	−1.9%	−4.2%	0.4%
Falls (unintentional)	2.71	1.03	−62.0%	0.511	−5.0%	−6.3%	−3.6%
Self-inflicted injury	4.53	1.45	−67.9%	0.633	−5.8%	−8.2%	−3.4%
Poisoning (self-inflicted)	4.12	1.50	−63.6%	0.832	−5.2%	−7.7%	−2.6%
Intentionally inflicted by another	5.10	2.39	−53.0%	0.920	−3.9%	−6.8%	−0.9%
Aboriginal, Male							
Unintentional injury	2.89	0.90	−68.9%	0.105	−6.0%	−6.9%	−5.0%
Transport vehicle (unintentional)	3.48	0.59	−83.2%	0.004	−9.0%	−10.6%	−7.3%
Medical/surgical mishaps	1.90	1.32	−30.6%	0.547	−1.9%	−5.0%	1.3%
Falls (unintentional)	2.68	0.93	−65.1%	0.320	−5.4%	−7.0%	−3.7%
Self-inflicted injury	4.63	1.46	−68.5%	0.903	−5.9%	−10.3%	−1.3%
Poisoning (self-inflicted)	3.27	1.51	−53.9%	0.728	−4.0%	−9.2%	1.5%
Intentionally inflicted by another	4.16	2.44	−41.3%	0.781	−2.8%	−6.0%	0.6%
Aboriginal, Female							
Unintentional injury	2.71	1.13	−58.1%	0.354	−4.5%	−5.7%	−3.2%
Transport vehicle (unintentional)	2.90	0.80	−72.5%	0.384	−6.6%	−8.9%	−4.2%
Medical/surgical mishaps	1.94	1.33	−31.7%	0.542	−2.0%	−5.3%	1.4%
Falls (unintentional)	2.77	1.20	−56.6%	0.890	−4.3%	−6.5%	−2.1%
Self-inflicted injury	4.49	1.45	−67.7%	0.617	−5.8%	−8.6%	−2.9%
Poisoning (self-inflicted)	4.39	1.50	−65.8%	0.686	−5.5%	−8.4%	−2.5%
Intentionally inflicted by another	9.06	2.16	−76.1%	0.824	−7.3%	−13.5%	−0.5%
BC							
Unintentional injury	1.75	0.64	−63.4%		−5.1%	−5.3%	−5.0%
Transport vehicle (unintentional)	2.06	0.54	−73.8%		−6.8%	−7.2%	−6.5%
Medical/surgical mishaps	1.27	0.71	−43.7%		−3.0%	−3.5%	−2.4%
Falls (unintentional)	1.60	0.67	−58.3%		−4.5%	−4.8%	−4.2%
Self-inflicted injury	1.59	0.58	−63.8%		−5.2%	−5.8%	−4.6%
Poisoning (self-inflicted)	1.54	0.59	−61.4%		−4.9%	−5.5%	−4.2%
Intentionally inflicted by another	1.47	0.71	−51.6%		−3.7%	−4.5%	−3.0%
BC, Male							
Unintentional injury	1.76	0.64	−63.6%		−5.2%	−5.4%	−5.0%
Transport vehicle (unintentional)	2.01	0.57	−71.8%		−6.5%	−6.9%	−6.0%
Medical/surgical mishaps	1.24	0.71	−42.8%		−2.9%	−3.6%	−2.2%
Falls (unintentional)	1.61	0.67	−58.6%		−4.5%	−4.9%	−4.1%
Self-inflicted injury	1.77	0.59	−66.5%		−5.6%	−6.7%	−4.5%
Poisoning (self-inflicted)	1.63	0.62	−61.9%		−5.0%	−6.2%	−3.7%
Intentionally inflicted by another	1.37	0.73	−46.5%		−3.2%	−4.0%	−2.5%
BC, Female							
Unintentional injury	1.73	0.64	−63.0%		−5.1%	−5.4%	−4.8%
Transport vehicle (unintentional)	2.14	0.47	−77.9%		−7.6%	−8.2%	−7.1%
Medical/surgical mishaps	1.30	0.72	−44.5%		−3.1%	−3.8%	−2.3%
Falls (unintentional)	1.58	0.67	−58.0%		-4.5%	−5.0%	−3.9%
Self-inflicted injury	1.52	0.57	−62.5%		−5.0%	−5.7%	−4.3%
Poisoning (self-inflicted)	1.50	0.58	−61.2%		−4.9%	−5.6%	−4.1%
Intentionally inflicted by another	2.01	0.56	−72.0%		−6.5%	−8.3%	−4.6%

Notes:

* probability (2-sided, z-test) that Ln((SRR 2009)/(SRR 1991)) Aboriginal = Ln((SRR 2009)/(SRR 1991)) BC.

Among Aboriginal females there was a reduction of 93.8% in SRR of hospitalization due to burn injury, compared to a reduction of 73.7% among the total BC female population (p = 0.038, 2-sided).

Among Aboriginal males, there was an 83.2% decrease in SRR of hospitalization due to injury from unintentional transportation vehicle collision, compared to 73.8% decrease among the total BC male population (p = 0.004, 2-sided).

Table 5 shows the reduction in SRR of hospitalization due to injuries intentionally inflicted by another person, for both genders, in both populations. In the Aboriginal population and the total BC population, females showed notably larger reductions in SRR (76.1% and 72.0% respectively) and than did males (41.3% and 46.5% respectively). In 1991, for both populations, the SRR was higher for females than for males, and for Aboriginal females more than double the SRR for Aboriginal males. By 2009, the larger decreases for females have brought the SRRs for females in both populations to below that of risk for males.

## Conclusions

This study observes substantial improvements in injury hospitalization risk among children and youth, under age 25 years, over the past two decades, for most major injury categories, among both the Aboriginal and total populations of BC, and for males and females within those populations. Because risk has declined in similar proportions for the Aboriginal and total BC populations, the disparity between the populations continues. Two exceptions are the significantly greater decrease in risk of burns among Aboriginal females compared to the female BC population and the decrease in risk of unintentional transport vehicle injury in the male Aboriginal population compared to the total BC male population.

Disparities in rates between the Aboriginal population and total BC population remain because of similarity in the proportional reductions among the two populations. However, since Aboriginal children and youth started at a much higher risk, in absolute terms, the gap between the two populations is shrinking. The parallel rates of decline between the two populations are consistent with another Canadian study of shorter duration on injury mortality rates for children (Harrop et al. 2007). Similar longitudinal data for adults show that by 2010, risk for injury is the same for the Aboriginal and total populations (George et al. 2015).

Differences between male and female risk vary by type and cause of injury. The over 70% declines in risks among females of intentionally inflicted injury by another, among both the Aboriginal and total BC populations is excellent news. Risk of injury caused by transport vehicles has decreased by an overwhelming 83% and 72% for the Aboriginal male population and for the total BC male population, respectively.

This study is unique in the period of time studied comparing the Aboriginal and total population for children and youth. Our study shows substantive decreases in hospitalization injury risks for children and youth in both populations over the 24-year period, 1986 to 2009. Risks in overall injury dropping by 69% for the Aboriginal population and by 66% for the total BC population are cause to celebrate. These results showing decreases are consistent with previously published results both for all ages of the Aboriginal and total population of BC (British Columbia Vital Statistics Agency 2004) and for overall child and youth injury hospitalization rates worldwide (Möller et al. 2015; Mytton et al. 2012; WHO World Health Organization 2002), and within Canada (Oliver and Kohen 2012; Alaghehbandan et al. 2010). All major types of injury and causes of injury showed decreases in rates over time for both populations, which is contrary with US studies which show levelling of rates or even increases (U.S. Department of Health and Human Services, 2014).

Limitations of the data pose some challenges for interpretation of results. First, hospitalizations due to injury can be influenced by availability of beds, medical practice and alternative options available for the patient and within the health care system, all of which may vary across regions and between Aboriginal and non-Aboriginal populations. Second, we used a restrictive operational definition of “Aboriginal” which is based on legal definitions (George et al. 2015) Standard definitions of Aboriginal do not exist for the purposes of inclusion in datasets. The definition used in these analyses is more likely to include children by definition of their parents’ status. As we have reported elsewhere (George et al. 2015), this inclusive definition is restrictive, but not so restrictive that it does not allow for generalizations.

An international review reported that Canadian studies of children and youth showed greater disparity between Aboriginal and non-Aboriginal children than studies elsewhere (Möller et al. 2015). The studies included in Möller et al. (2015)’s review were from countries (USA, Australia and New Zealand) with similar colonial histories and socioeconomic status as Canada; therefore, it is curious that Canadian studies show greater disparity. Across 39 studies, rate ratios for morbidity rates ranged from 1.2 to 2.3 and mortality rates from 1.8 to 8.2 (Möller et al. 2015). The four Canadian studies noted for having higher disparity rates included two mortality studies (Peters et al. 2013; Oliver et al. 2012), two examining hospitalization rates (Oliver and Kohen, 2012) and one showing both injury outcomes (Alaghehbandan et al. 2010). Each study, along with another Canadian study (Harrop et al. 2007) on mortality rates which was not specifically noted as having larger disparity, used area-based census as a method of identifying Aboriginal populations. Four Canadian studies (Harrop et al. 2007, Peters et al. 2013, Oliver et al. 2012, Alaghehbandan et al. 2010) comparing mortality rates showed rates 3 times to 8 times higher risk for the Aboriginal children and youth than their comparative populations. Two studies (Oliver and Kohen, 2012; Harrop et al. 2007) comparing hospitalization rates showed Aboriginal children and youth to have approximately double the risk of their counterparts.

A contribution of this study is the method of identification of the Aboriginal population within the population-based dataset, rather than using area-based identification. This method of analysis has not been used previously in injury studies of Canadian children and youth. Secondly, the extensive time period (24 years for types of injury and 19 years for causes of injury) provide opportunities to describe changes in disparity between the Aboriginal and total populations and between males and females.

Our results, using a refined method of identifying the Aboriginal population within the total population compared to the area-based identification used in the studies noted above, continue to show disproportion of injury hospitalizations for Aboriginal children and youth. For all injuries, the SRRs overall were 1.9 (1986) and 1.8 (2009), for males 1.7 (1986) and 1.6 (2009) and for females 2.3 (1986) and 2.0 (2009). In all categories of injuries, only burns for Aboriginal females show lower rates compared to the total female population. Disparity exists for all categories of injury, with the exception of injuries caused by transport vehicles with similar rates for Aboriginal males and total BC males (0.59 compared to 0.57 respectively) in 2009.

Our results show some smaller gaps between rates for the Aboriginal and total BC populations compared with the other Canadian studies (Peters et al. 2013; Oliver et al. 2012; Alaghehbandan et al. 2010; Harrop et al. 2007). Four possible explanations for this are that our method of identification of the Aboriginal population provides more accurate results, differences in dates of the studies provide non-comparable results, our specific populations show differences in results such that the gap is smaller in BC than elsewhere, or that the gap between the Aboriginal and total populations is closing in Canada, albeit slowly.

While we are encouraged by the significant decreases over time in injury risk for Aboriginal children and youth, we note that disparities continue for many specific types and categories of injuries. It is clear that more work is needed in Canada to reduce health inequities for all, and especially in the area of injury among children. While our results indicate good news, they should not lead to complacency with respect to prevention initiatives, since injuries are associated with consequent economic and social burdens (SmartRisk 2009; Chandran et al. 2010).

From a public health perspective, these results should lead to further inquiry about the causes of high rates, sector by sector. Population-level results do not take into account individual differences or causation. Taking a population-based public health approach could lead to disparity (Frohlich and Potvin 2008a), depending on proximal and distal causes. Further, interventions should be participatory (Frohlich and Potvin 2008b), so that by nature they differ according to the population by whom they are designed. Much has been written about disparities in Canada identifying determinants, including Aboriginal status (Adelson 2005; Frohlich et al. 2006; George et al. 2013). Sustainable solutions require health promotion initiatives and policy that consider individual-level, community-level and population-level realities in order to reduce injury rates for all (Frohlich and Potvin, 2008b; Richmond and Ross, 2009; Rose, 1992).
